# First Report of *Phytophthora mengei* Causing Root Rot and Canker in Avocado (*Persea americana*) in Michoacán, Mexico

**DOI:** 10.3390/microorganisms13071471

**Published:** 2025-06-24

**Authors:** Alejandra Mondragón-Flores, Alejandro Soto-Plancarte, Gerardo Rodríguez-Alvarado, Patricia Manosalva, Salvador Ochoa-Ascencio, Benjamin Hoyt, Nuria Gómez-Dorantes, Sylvia Patricia Fernández-Pavía

**Affiliations:** 1 Campo Experimental Uruapan, Instituto Nacional de Investigaciones Forestales, Agrícolas y Pecuarias (INIFAP), Av. Latinoamericana No. 1101, Revolución, Uruapan 60150, Michoacán, Mexico; mondragon.flores@gmail.com; 2 Instituto de Investigaciones Agropecuarias y Forestales, Universidad Michoacana de San Nicolás de Hidalgo (UMSNH), Km 9.5 Carretera Morelia-Zinapeécuaro, Tariémbaro 58880, Michoacán, Mexico; alexsotoppv@gmail.com (A.S.-P.); gra.labpv@gmail.com (G.R.-A.); nuria.gomez@umich.mx (N.G.-D.); 3 Department of Microbiology and Plant Pathology, University of California, Riverside, 900 University Avenue, Riverside, CA 92521, USA; pmanosal@ucr.edu (P.M.); benjamin.hoyt@email.ucr.edu (B.H.); 4 Facultad de Agrobiología, Universidad Michoacana de San Nicolás de Hidalgo (UMSNH), Paseo Lázaro Cárdenas No. 2290, Jardines del Cupatitzio, Uruapan 60170, Michoacán, Mexico; salvador.ochoa@umich.mx

**Keywords:** oomycete, avocado diseases, pathogenicity

## Abstract

Mexico is the world’s leading producer of avocado (*Persea americana*); however, its productivity is threatened by various diseases, especially root rot caused by *Phytophthora*. While *P*. *cinnamomi* is the most commonly reported species worldwide, this study identified *P*. *mengei* for the first time as a causal agent of root rot and trunk canker in avocado orchards in the state of Michoacán, México. The morphological and molecular characterization of four isolates (three from canker and one from root rot) confirmed their identity: semi-papillate sporangia and plerotic oospores with paragynous antheridia, with sequence identities of 99.87% (*ITS*) and 100% (*COI*) with type sequences of *P*. *mengei*. Pathogenicity tests demonstrated the ability to infect roots, stems, and fruits, although with a low reisolation percentage in roots (10%), suggesting an opportunistic pathogen behavior. Sensitivity tests to potassium phosphite (EC_50_ of 3.67 μg/mL^−1^ a.i.) and metalaxyl-M (0.737 μg/mL^−1^ a.i.) revealed possible limitations for chemical control. These findings position *P*. *mengei* as an emerging pathogen with important implications for integrated crop management. To the best of our knowledge, this is the first report of *P*. *mengei* causing root rot and trunk canker in avocado in Michoacán, Mexico.

## 1. Introduction

Avocado is a fruit with wide acceptance worldwide and has been broadly commercialized due to its high nutritional value; it is a source of lipids and proteins with important health benefits [1]. In 2023, avocado production in Mexico reached 2.65 million tons, with more than 72% originating from the state of Michoacán [2]. One of the main limitations to avocado cultivation is root rot caused by *Phytophthora cinnamomi* [3,4], which destroys secondary roots and spreads through the vascular bundles, causing foliar chlorosis, defoliation, dieback of branches, and trunk cankers at the base of the tree [5,6]. Additionally, *P. citricola* (currently known as *P. mengei*) has been reported to cause root rot and canker in avocado [7,8,9]. Trees with wounds, water stress, excessive salinity, low temperatures, or those already infected with *P. cinnamomi* have been observed to be more susceptible to *P. citricola* [10], which, unlike *P. cinnamomi*, has a more limited host range [11].

The first report of *P*. *citricola* in avocado was made by Zentmyer et al. [12]. Later, Oudemans et al. [13] analyzed the isoenzymes of *P*. *citricola* isolates—including some obtained from avocado (fruit, root, and canker) and other hosts in different countries—and concluded that it was a species complex. In 2009, Hong et al. [9] formally separated a subgroup of *P*. *citricola* associated with avocado, reclassifying it as *Phytophthora mengei* sp. nov. In the study by Oudemans et al. [13], three isolates of *P. citricola* from avocado of Mexico were also included (ATCC52230, P0513, and P3710), isolated from black fruit rot, and isolate P7127 [7,14], which grouped with isolates from avocado in California, suggesting they could correspond to *P*. *mengei*. However, it was not until 2010 that Bezuidenhout et al. [15], analyzing isolates from the *P*. *citricola* complex from various hosts and countries, reclassified the isolate ATCC52230-P0513 (obtained from fruit rot in Villa Guerrero, State of Mexico) as *P*. *mengei*, after analyzing five genetic regions (*ITS, β-tubulin, EF-1α, NADH*, and *COX1*). This reclassification represents the first mention of *P*. *mengei* in Mexico.

In addition, Abad et al. [11] indicated that *P*. *mengei* was restricted to California, USA, but other authors have also reported it in Guatemala and Mexico [16,17,18]. However, Jung et al. [18] clarified that an isolate from Guatemala (WPC P1165) previously classified as *P*. *mengei* actually corresponded to a different taxon, designated as *P*. *mengei*-like, a sister species possibly endemic to Guatemala. Outside the American continent, *P*. *mengei* has only been reported in Australia, in *Vigna unguiculata*, a host different from avocado [19].

In Mexico, aside from *P*. *cinnamomi*, other *Phytophthora* species associated with avocado diseases have been reported, such as *P*. *heveae* (causing trunk canker and basal fruit rot) [20,21] and *P*. *parasitica* (synonym *P*. *nicotianae*) [20]. *P*. *boehmeriae* was mistakenly reported as a pathogen of avocado in Mexico [22,23], a misclassification later corrected [11,24]. Therefore, as stated in [11], DNA barcoding using the *ITS* and *COI* genes is a valid approach for identifying *Phytophthora*, information that is essential for accurately describing the *Phytophthora* species present in avocado crops in Mexico.

The continued expansion of avocado cultivation in Michoacán has increased the incidence and diversity of diseases affecting this crop. Given the economic importance of avocado in the region, it is crucial to characterize the causal agents, as losses in yield and quality can be significant (according to information gathered). During sampling carried out in commercial orchards in the municipalities of Villa Madero and Zitácuaro, *Phytophthora* strains were isolated from roots with rot and trunks with canker that did not morphologically match the known species in the region. Therefore, the objectives of this study were (1) to morphologically and molecularly characterize these isolates, (2) to demonstrate their pathogenicity in avocado, and (3) to evaluate their sensitivity to potassium phosphite and metalaxyl-M.

## 2. Materials and Methods

### 2.1. Sampling and Isolation

During the years 2019 and 2023, sampling was conducted in commercial ‘Hass’ avocado orchards in the municipalities of Zitácuaro and Villa Madero, Michoacán. Roots and stem tissue were collected from trees showing symptoms of root rot (Zitácuaro) and trunk canker (Villa Madero). Isolation from the samples was carried out following the protocol described by Mondragón-Flores et al. [25]. Briefly, lateral roots, as well as bark and cambium tissues, were washed with running water, dried, cut into fragments of approximately 1 cm, and placed into embedding cassettes (Fisher™ [Tulare, CA, USA]). Roots were disinfested with 2% commercial bleach for 45 s and then rinsed three times with sterile distilled water (dH_2_O). After drying with sterile absorbent paper, they were plated onto Petri dishes containing NARPH-V8 selective medium (natamycin 0.02 g/L, ampicillin 0.27 g/L, rifampicin 0.01 g/L, PCNB 0.10 g/L, and hymexazol 0.075 g/L). The bark and cambium fragments were plated directly onto NARPH-V8 medium after washing and drying. The plates were incubated at 25 °C in the dark for 48 h until the characteristic coenocytic mycelial growth of *Phytophthora* was observed. Mycelium was transferred to V8-A medium (15 g of agar, 3 g of CaCO_3_, 160 mL of Campbell’s™ V8 juice, 840 mL of distilled water). Later, pure cultures were obtained using the hyphal tip method on 1.5% water agar [26] and grown on V8-A medium at 25 °C for morphological characterization.

### 2.2. Morphological Characterization

The isolates were grown for six days on V8-A medium. To induce sporangia formation, agar blocks with approximately 1 cm^2^ of mycelium were cut, and sterile distilled water was added up to the edge of the agar. These were incubated at 25 °C under continuous white light, renewing the water daily for four days. Structures were observed and 30 sporangia were measured using a Leica DME microscope (Durham, NC, USA) (40× objective), evaluating sporangium shape and sporangiophore morphology.

The canker isolates, which exhibited slow growth at 25 °C and did not form oospores, were grown on corn meal agar (CMA), potato dextrose agar (PDA), V8-A, rye meal agar (RMA), and green bean–squash agar (GBSA) at 20 °C in darkness [27,28] to induce sexual structure formation. Once formed, 30 oospores were measured, and the type of antheridium was determined.

### 2.3. Molecular Characterization

The isolates were cultivated on GBSA medium covered with a sterile cellophane disk at 25 °C for five days. The mycelium was collected, dehydrated at 37 °C for 24 h, and ground in liquid nitrogen. Genomic DNA was extracted using the cetyltrimethylammonium bromide (CTAB) method (Sigma-Aldrich, St. Louis, MO, USA) [27] and quantified using a Varioskan ^®^ Flash Thermo Fisher Scientific (Waltham, MA, USA).

The *ITS* region was amplified using primers *ITS*6 and *ITS*4 [29,30], and the mitochondrial *COI* gene was amplified with primers OomCox1-Levup and Fm85mod [31]. PCR reactions were performed on an Eppendorf, Mastercycler Gradient thermocycler (Eppendorf, Hauppauge, NY, USA) using 3 μL of DNA (12 ng/μL^−1^), 6.25 μL of Master Mix (GoTaq™ Hot Start, Promega, Madison, WI, USA), 0.0675 μL of each primer (100 pmol/μL^−1^), and 3.615 μL of molecular-biology-grade water. The conditions were initial denaturation at 94 °C for 2 min; 35 cycles at 94 °C for 1 min, 60 °C (*ITS*) or 50 °C (*COI*) for 1 min, 72 °C for 2 min; and a final extension at 72 °C for 10 min. These two genes were selected to barcode the DNA of the isolates, since they are considered the most important for distinguishing *Phytophthora* species [11,31].

The amplified products were visualized on a 1.5% agarose gel stained with ethidium bromide (5 μg/mL^−1^) under UV light (High-Performance UV Transilluminator UVP, TFML-26, Upland, CA, USA). Amplicons were purified with a Wizard SV Gel and PCR Clean-Up System kit (Promega, Madison, WI, USA) and sent to Macrogen, South Korea, for the sequencing of both strands. Consensus sequences were generated using the gap5 and Pregap4 programs from the Staden package 2.0b11 [32] and analyzed with BLASTn on NCBI (https://blast.ncbi.nlm.nih.gov/Blast.cgi (accessed on 30 September 2024)), comparing them with type sequences according to Abad et al. [11].

### 2.4. Phylogenetic Analysis

To confirm isolates identity using the type sequences of *P*. *mengei* (CPHST BL 31), a maximum likelihood phylogenetic analysis was performed with concatenated *ITS* and *COI* sequences. Sequences from various *Phytophthora* species were downloaded from NCBI GenBank (listed as Supplementary Material Table S1) and aligned using MEGA 11 [33]. The phylogenetic tree was constructed using IQ-TREE in CIPRES [34] with 1000 bootstrap replicates and visualized in iTOL 7.1.1 [35].

### 2.5. Pathogenicity Tests on Avocado Plants with Root Isolate

The root isolate PV52 was grown on 10% V8-A medium for five days at 25 °C in darkness. The medium containing mycelium was cut into ~1 cm^2^ blocks; the plates were flooded with Zentmyer solution [36] (1.64 g of Ca(NO_3_)_2_, 0.05 g of KNO_3_, and 0.48 g of MgSO_4_ dissolved in 1 L of distilled water, sterilized for 20 min at 15 psi) and exposed to continuous white light. After 48 h, the agar blocks were gently rubbed with a sterile swab to release sporangia, and a suspension was prepared and quantified in a Neubauer chamber [27]. Five young ‘Hass’ avocado plants (12–14 true leaves) from a nursery in Uruapan, Michoacán, were inoculated by immersing their roots in 4 L of the suspension (660,000 sporangia) for three hours. Three control plants were immersed in sterile distilled water. Plants were then transplanted into pots and maintained in a greenhouse. Reisolations were carried out on NARPH-V8 medium 20 days post-inoculation, following the protocol previously described.

### 2.6. Pathogenicity Tests with Canker Isolate on Rootstocks

Three 13-month-old ‘Hass’ avocado plants grafted onto *P. americana* var. *drymifolia* rootstocks were inoculated with isolate PV51. Prior to inoculation, stems were surface-sterilized with 70% ethanol, and two vertical and one horizontal (~2 cm) cuts were made in the bark, inserting a V8-A medium disk containing mycelium (10 days, 20 °C, darkness) between the bark and the xylem. In control plants, disks without mycelium were placed. The inoculation sites were sealed with parafilm. Inoculation was performed in the afternoon at 27 °C. Greenhouse temperatures during September 2024 ranged from 12 to 39 °C and in October from 6 to 43 °C. Symptoms were monitored weekly. Reproductive structures were analyzed from lesions with exudate, collected with tape, and placed on microscope slides.

### 2.7. Pathogenicity Tests on Avocado Fruits

Physiologically mature ‘Hass’ fruits were used. For each isolate, three fruits were inoculated, and three fruits served as controls. Fruits were washed, surface-sterilized with 70% ethanol, and wounded with a scalpel (~5 mm). Agar disks with mycelium (5 mm, 6-day cultures) were placed over the wounds. Fruits were kept in a humid chamber at 25 °C for four days. Symptoms were recorded, and reisolations from necrotic zones and healthy exocarp were conducted on NARPH-V8 medium.

### 2.8. Fungicide Sensitivity

To assess sensitivity to potassium phosphite (PP) (Nutriphite Plus Magnum 40%, Gowan™) [Cary, NC, USA] and metalaxyl-M (MT-M) (RidomilGold™ 480SL) [Greensboro, NC, USA], isolate PV52 was selected. It was exposed to various concentrations of both compounds, following the protocol described by Mondragón-Flores et al. [25]. The concentrations tested for PP were 0, 5, 10, 25, 50, 100, 300, and 600 μg/mL^−1^ a.i., and for MT-M, 0, 0.05, 0.15, 0.5, 1, 3, and 5 μg/mL^−1^ a.i.

The effective median concentration (EC_50_) for each fungicide was determined using the agar dilution method described by Gray et al. [37], and the values were calculated using a probit regression with R software version 4.3.2 and the ecotox library version 1.4.4, employing the natural logarithm of the concentrations [38].

Four-day-old mycelial disks were placed at the center of Petri dishes containing 10% clarified V8 agar (cV8-A) supplemented with the fungicide concentrations. Plates were arranged in a completely randomized design. Mycelial growth was measured three days post-inoculation and is expressed as percentage of growth inhibition, as described by Hu et al. [39]. Experiments were performed in duplicate.

## 3. Results

### 3.1. Sampling and Isolation

**Figure 1:**
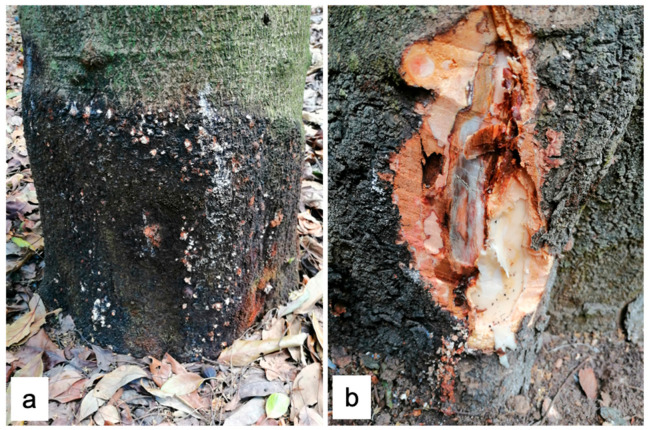
Trunk canker observed in Villa Madero, Michoacán. (**a**) Canker in the rootstock, with whitish and occasionally reddish exudates, without bark detachment; (**b**) cross-section showing reddish-brown necrosis in the bark and cambium at the base of the stem as well as bark cracking.

Four isolates were obtained from 20 processed samples. From the municipality of Zitácuaro, a root isolate (PV52) was obtained from a 30-year-old rainfed tree located in the locality of Carpinteros (19°49′05″ N, 100°31′58″ W) at an altitude of 2176 m above sea level. In Villa Madero, three isolates (PV49, PV50, and PV51) were obtained from canker tissue in different plants from an orchard of 15-year-old trees located at 19°24′53″ N, 101°18′44″ W at 2269 m above sea level. Both orchards had clay soils (acrisol), and the trees were of the ‘Hass’ variety. Canker lesions were located at the base of the rootstock, in contact with the soil, without bark detachment (Figure 1). The external lesion sizes caused by isolates PV49, PV50, and PV51 were approximately 40 × 25 cm, 50 × 40 cm, and 50 × 15 cm, respectively.

### 3.2. Morphological Characterization

The observed sporangia were semi-papillate, arranged in simple sympodia, with some presenting double papilla. Sporangia shapes included ovoid, pyriform, ellipsoidal, elongated, and irregular forms, and size ranged from 32.5–77.5 × 17.5–37.5 μm, with a persistent pedicel.

**Figure 2:**
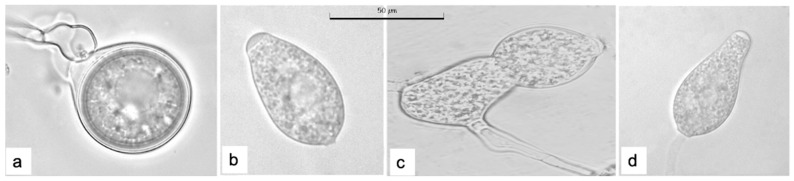
Structures of *Phytophthora mengei*. (**a**) Plerotic oospore with paragynous antheridium; (**b**) ovoid, semi-papillate sporangium; (**c**) irregular-shaped sporangium; (**d**) pyriform, semi-papillate sporangium.

The isolates incubated at 20 °C produced oospores on V8-A and RMA media. Oospores were smooth, predominantly plerotic, measuring 17.5–25.0 × 17.5–27.5 μm, with some aplerotic oospores and asymmetrically capitate paragynous antheridia (Figure 2). No oospore production was observed on CMA and PDA media.

### 3.3. Molecular Characterization

The BLASTn analysis of the *ITS* and *COI* sequences showed identities of 99.87% (100% coverage) and 99.04% (98% coverage), respectively, with the type sequences of *Phytophthora mengei* (CPHST BL 31), grouped within clade 2b [11]. The sequences were deposited in GenBank. The accession numbers for the isolates for *ITS* and *COI* are as follows: PV49 (OR479677, OR493518), PV50 (OR479678, OR493519) PV51 (OR479679, OR493520), PV52 (OR479680, OR493521).

### 3.4. Phylogenetic Analysis

**Figure 3:**
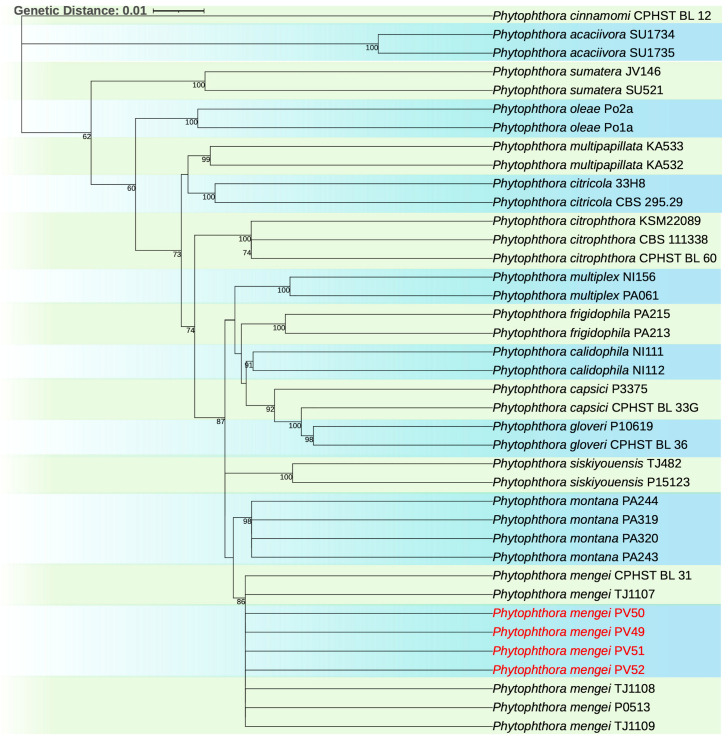
Maximum likelihood concatenated phylogenetic tree created with *ITS* and mitochondrial *COI* gene sequences of *Phytophthora* spp. in clade 2, rooted with the type sequence of *Phytophthora cinnamomi* CPHST BL 12 as the outgroup. New isolates found in this study are highlighted in red.

In the phylogenetic analysis, the concatenated tree with the *ITS* and *COI* sequences showed that isolates PV49, PV50, PV51, and PV52 grouped with the type sequence of *P*. *mengei* (CPHST BL 31). The tree topologies and bootstrap values are shown in Figure 3.

### 3.5. Pathogenicity Tests on Avocado Plants with Root Isolate

**Figure 4:**
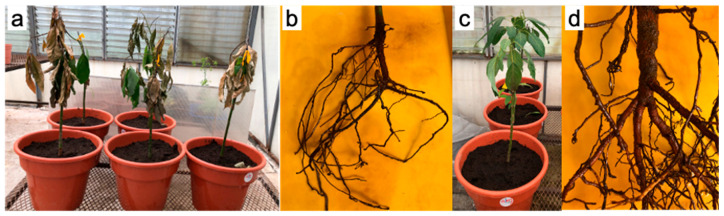
Pathogenicity tests of *P*. *mengei* on avocado plants. (**a**) Plants with defoliation and dieback at 20 dpi; (**b**) necrotic root without feeder roots from an inoculated plant; (**c**) control plants without symptoms; (**d**) control plant roots without symptoms.

Isolate PV52 was pathogenic. Six days after inoculation (dpi), plants showed chlorosis, defoliation, and dieback. By 20 dpi, most leaves had fallen. Roots showed necrosis, bark detachment, and the absence of feeder roots. The control plants did not develop symptoms. The pathogen was reisolated from inoculated plants (Figure 4). The reisolation frequency was 10%.

### 3.6. Pathogenicity Tests with Canker Isolate on Rootstocks

**Figure 5:**
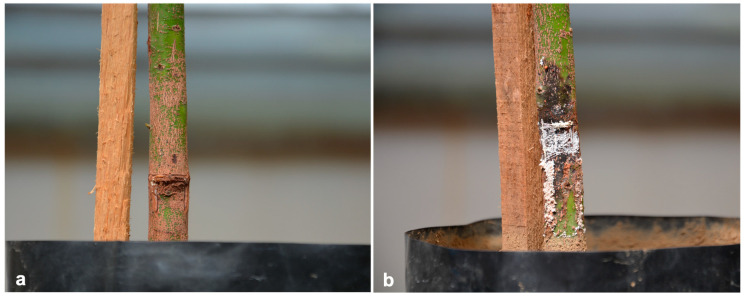
Pathogenicity tests with trunk canker isolate (PV51) on ‘Hass’ avocado seedling rootstocks. (**a**) Control plant inoculated with a V8-A medium disk; (**b**) plant inoculated with a V8-A mycelium disk of *P*. *mengei*, showing necrosis and reddish and whitish exudates.

Isolate PV51 was pathogenic. The inoculated rootstocks showed the first necrosis symptoms at 4 dpi; by 15 dpi, reddish and whitish exudates were observed. At 32 dpi, lesions reached 7 cm in size, predominantly with whitish exudate and minor reddish exudate (Figure 5). Oospores were observed in the samples from the exudates. The control plants showed no symptoms. The inoculated pathogen was reisolated from symptomatic plants.

### 3.7. Pathogenicity Tests on Avocado Fruits

**Figure 6:**
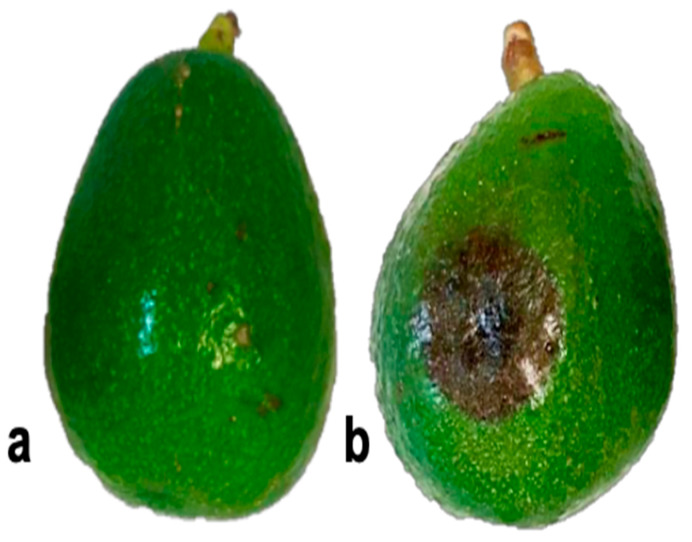
Pathogenicity tests with trunk canker isolate (PV51) on ‘Hass’ avocado fruits. (**a**) Control fruit without symptoms; (**b**) inoculated fruit with symptoms (5 dpi) at an artificial wound.

Three isolates (PV49, PV51, and PV52) were pathogenic in fruit. Isolate PV50 did not cause infection. At 4 dpi, infected fruits showed dark brown, circular lesions. The control fruits did not develop symptoms (Figure 6). *P*. *mengei* was reisolated at 5 dpi, showing slow growth on NARPH-V8 medium at 25 °C in darkness. The morphological characteristics of the reisolated pathogen matched the inoculated isolate.

### 3.8. Fungicide Sensitivity

**Figure 7:**
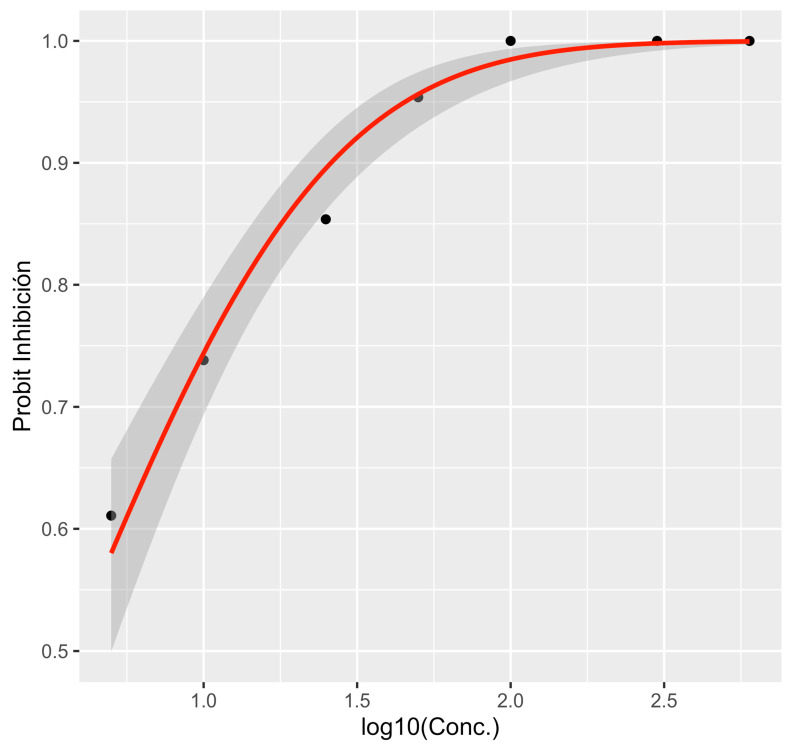
Regression plot showing the dose–response curve of potassium phosphite. The gray band shows the 95% confidence interval.

**Figure 8:**
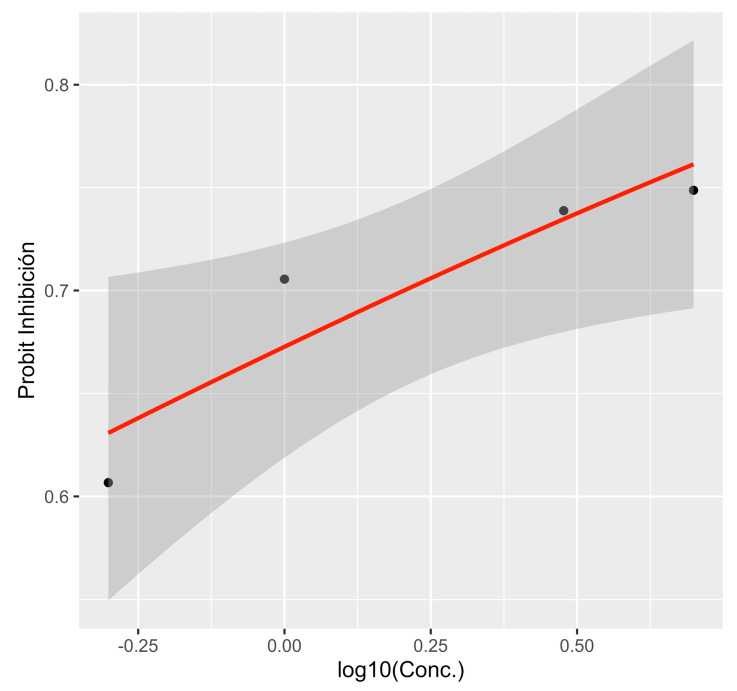
Regression plot showing the dose–response curve of metalaxyl-M. The gray band shows the 95% confidence interval.

Variance analyses showed significant differences (*p* < 0.001) in mycelial growth and inhibition depending on PP and MT-M concentrations. For potassium phosphite (PP), the average mycelial growth was 5 mm, with 77.0% inhibition. Concentrations of 100, 300, and 600 μg/mL^−1^ a.i. inhibited growth by 100% and were statistically equal (Table 1). For metalaxyl-M (MT-M), the average growth was 11.6 mm and 41.6% inhibition. At 3 and 5 μg/mL^−1^ a.i., the growth was inhibited by over 70% (Table 1). The EC_50_ values were 3.67 μg/mL^−1^ a.i. for PP (probit regression equation (*p*) = −0.852 + 1.51 × concentration) and 0.737 μg/mL^−1^ a.i. for MT-M (probit regression equation (*p*) = 0.165 + 1.24 × concentration (Figure 7 and Figure 8).

## 4. Discussion

Since Michoacán is the world’s leading producer of avocados, the recent detection of *P. mengei* in avocado orchards is of great significance. The results of the present study confirm for the first time the presence of *P. mengei* affecting avocado in Michoacán, Mexico, a finding with critical implications for the avocado industry, considering that this state produces more than 72% of the country’s avocados [2].

Unlike previous studies that identified *P*. *mengei* (formerly *P. citricola*) only in fruits or regions outside the world’s most important avocado production area [14,40,41], our results demonstrate the pathogenic capability of this species in the roots, stems, and fruits, suggesting a previously unrecognized phytosanitary risk.

This represents the first report, supported by morphological, molecular, and pathogenicity evidence under controlled conditions, confirming the presence of *P*. *mengei* in Michoacán’s avocado-producing area. Reports on the presence of *P*. *citricola* (now *P*. *mengei*) in Mexico refer mainly to the State of Mexico in the 1980s (on fruits) [14,42] and trunk canker in Puebla during the 1990s [41]. It is likely that *P*. *mengei* arrived in Michoacán orchards through infected nursery plants.

The isolates analyzed both morphologically and molecularly matched those previously reported as *P*. *mengei* [9,11]. However, they present differences in optimal growth temperature. While the reported optimal temperature is 25 °C [11], in this study, very slow development was observed at 25 °C, while greater colony growth was seen at 20 °C on V8-A, RMA, and GBSA media. This finding differs from what Bezuidenhout et al. [15] reported, who observed unstable growth of the Mexican isolate and no oospore production on carrot–agar medium at 20 °C in the dark. In the present study, no oospore formation was observed in CMA and PDA media at 20 °C either; additionally, the root isolate did not produce oospores on RMA, while all isolates produced oospores on V8-A and GBSA media. This suggests a difference between the root and canker isolates. 

The morphological characteristics observed in the sporangia and oospores, as well as the presence of irregularly shaped sporangia in the isolates recovered in this study, are consistent with previous descriptions. The sporangia size (50 × 32.6 μm) slightly differs from the dimensions reported by Hong et al. [9] (62.7 × 35.2 μm), which could be explained by the differences in growth conditions and culture media used.

The molecular analyses confirmed the identity of the isolates as *P*. *mengei*, grouped within clade 2b, consistent with Hong et al. [9]. The morphological similarity between *P*. *mengei* and other clade 2b species has led to misdiagnoses in the past [13,19]. Our phylogenetic analyses based on concatenated sequences (*ITS* + *COI*) help clarify this confusion, locating the Mexican isolates firmly within the *P*. *mengei* taxon (type sequence CPHST BL 31), emphasizing the importance of implementing standardized molecular techniques for accurate diagnosis.

In the fungicide sensitivity tests, EC_50_ values of 3.67 μg/mL^−1^ a.i. (for PP) and 0.737 μg/mL^−1^ a.i. (for MT-M) were obtained for *P*. *mengei*. In previous studies, *P*. *cinnamomi* exhibited EC_50_ values of 24.62 and 0.215 μg/mL^−1^ for PP and MT-M, respectively [43], indicating that *P*. *mengei* is more sensitive to PP but more tolerant to metalaxyl-M (mefenoxam) compared to *P. cinnamomi*. Some authors suggested that tolerance in *P*. *mengei* populations to this fungicide could be an inherent trait of the species rather than a result of selection pressure, as several isolates had not been previously exposed to the fungicide [44]. The sensitivity results of *P. mengei* to both fungicides suggest their potential use in the control of avocado trunk canker within sustainable management programs through the strategic alternation of active compounds.

In the United States, PP has been used more frequently to control root rot than metalaxyl-M due to its effectiveness and lower cost [38,45]. This coincides with the current practices of Michoacán producers (according to information gathered), who apply PP by injection, a practice that creates wounds and can facilitate the entry of wood-pathogenic fungi. It is recommended to apply PP via irrigation systems or drenching. However, it must be considered that phosphite metabolizes to phosphonic acid, which can accumulate in fruits and affect exports to the European Union if maximum residue limits are exceeded.

Possibly, frequent PP applications have controlled *P. mengei* populations due to their fungicide sensitivity, which may partly explain why its presence had not been detected earlier in Michoacán. Alternatively, underdiagnosis due to isolation difficulties with *Phytophthora* [5], as shown by the low isolation percentage from infected roots in this study, may be responsible. Additionally, the low reisolation rate suggests asymptomatic infections.

Regarding pathogenicity tests, *P*. *mengei* caused rot in fruits and plants, developing symptoms such as defoliation, dieback, and root rot. However, in reisolation, the pathogen was 100% recovered from fruits but only 10% from roots. This suggests that *P*. *mengei*, although a weak root pathogen compared to *P*. *cinnamomi* [12], can cause significant fruit rot and more aggressive cankers on stems, facilitating its spread via infected nursery plant material to new cultivation areas.

Factors that favor disease development include excessive orchard shading, high relative humidity, clay soils, trees older than 10 years, and tree stress conditions. Cankers caused by *P*. *citricola* have been observed under similar conditions in California [8,10], which match the conditions prevalent in the sampled orchards. The presence of *Phytophthora* in new regions of Michoacán, such as Atécuaro and Zitácuaro [26], results from the expansion of avocado cultivation into areas that favor pathogen proliferation or where suboptimal conditions stress the plants. Furthermore, asymptomatic nursery plants could serve as vectors for *Phytophthora* dispersal into previously uninfected regions. The optimal development of *P*. *mengei* at 20 °C suggests adaptation to cooler microclimates at higher elevations in Michoacán.

For precise diagnosis, it is crucial to consider that cankers caused by *P. cinnamomi* are restricted to the trunk base, whereas those caused by *P*. *mengei* invade structural roots, the trunk, and branches, reaching up to three meters high [23]. According to Oudemans et al. [13], *P*. *mengei* may have been introduced into California through avocado plants originating from Mexico or Guatemala.

The accelerated expansion of avocado cultivation into marginal areas in Mexico means that *P*. *mengei* represents an emerging threat. Therefore, active epidemiological monitoring is necessary in orchards with clay soils and water stress, conditions favorable to *P*. *mengei* establishment. More extensive sampling is essential, given avocado’s economic relevance, to determine the *Phytophthora* species present, associated symptoms, and conditions promoting disease development, including for *P*. *mengei*, to establish appropriate management strategies.

## 5. Conclusions

*Phytophthora mengei* is an emerging pathogen with the potential to cause significant losses due to its ability to induce cankers, as well as root and fruit rot. This study not only represents the first report of *P. mengei* in avocado in Michoacán but also reveals critical shortcomings in the phytosanitary management of root rot and trunk canker in avocado.

The presence of both *P. mengei* and *P. cinnamomi* in Michoacán constitutes a pathogenic complex with a high potential to negatively affect the production, profitability, and long-term sustainability of avocado cultivation. Therefore, it is strongly recommended that commercial avocado nurseries and orchards be regularly monitored for the presence of *Phytophthora*.

## Figures and Tables

**Table 1:** Mycelial growth and inhibition of *P. mengei* at different concentrations of potassium phosphite and metalaxyl-M.

Potassium Phosphite					Metalaxyl-M				
Concentration (μg/mL^−1^)	Growth (mm)		Inhibition (%)		Concentration (μg/mL^−1^)	Growth (mm)		Inhibition (%)	
0	21.7	a *	0	a	0	19.8	a	0	a
5	8.5	b	61.0	b	0.05	19.5	ab	1.7	a
10	5.7	b	73.9	c	0.15	17.3	b	12.5	b
25	3.2	d	85.4	d	0.5	7.8	c	60.4	d
50	1.0	e	95.4	e	1	6.3	cd	68.1	dc
100	0	f	100	f	3	5.16	d	73.9	c
300	0	f	100	f	5	5.0	d	74.9	c
600	0	f	100	f					

* Values with the same letter are statistically equal in rows and columns (Tukey’s 0.05).

## Data Availability

The original contributions presented in this study are included in the article/Supplementary Material. Further inquiries can be directed to the corresponding author.

## Supplementary Materials

The following supporting information can be downloaded at: https://www.mdpi.com/article/10.3390/microorganisms13071471/s1, Table S1: Accession numbers of sequences of *Phytophthora* species used to construct the phylogenetic tree.
